# Educating patients with upper limb dysfunction on self‐adjustment of the CPAP/NPPV mask: A case series

**DOI:** 10.1002/rcr2.1232

**Published:** 2023-10-12

**Authors:** Tomoko Kuroda, Kazushige Ichikawa, Satoshi Hinata, Kaoru Chiba, Ken Okabayashi, Yuko Asato, Hiromi Aono, Kazuyoshi Marumo

**Affiliations:** ^1^ Nursing Department Tokyo Metropolitan Police Hospital Tokyo Japan; ^2^ Rehabilitation Department Tokyo Metropolitan Police Hospital Tokyo Japan; ^3^ Respirology Division, Department of Internal Medicine Tokyo Metropolitan Police Hospital Tokyo Japan; ^4^ Hoken Kaikan Clinic Tokyo Health Service Association Tokyo Japan

**Keywords:** mask‐fitting, self‐adjusting, upper limb dysfunction

## Abstract

We share our experiences of instructing three patients with severe upper limb dysfunction on how to self‐adjust CPAP/NPPV masks. In Case 1, we simplified the procedure by suturing a part of the headband as the left forearm was amputated. In Case 2, the patient had congenitally short limbs with short stature; thus, we provided an additional belt to the headband to maintain the headband's configuration while wearing the mask. In Case 3, the patient had left hemiplegia due to stroke and, repetitive coaching was conducted during the recovery phase rehabilitation program. Difficulties with self‐adjusting NPPV/CPAP masks can occur whenever there is limited hand mobility above the head, including upper limb dysfunction. Simplifying procedures and providing sufficient time for instruction could help achieve independence. There have been no previous reports describing similar training details. We believe that sharing this knowledge will be helpful to both patients and healthcare professionals.

## Graphical abstract

We provided instruction on CPAP/NPPV mask self‐adjustment to three patients with severe upper extremity dysfunction, offering a simple procedure and support tailored to their individual needs. This approach allows for independence in mask adjustment despite limited upper extremity mobility and benefits both patients and health care providers by sharing knowledge.

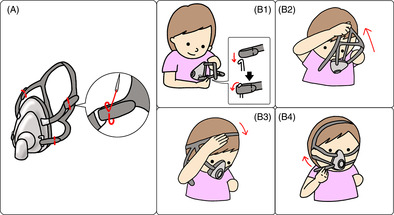

## INTRODUCTION

Non‐invasive positive pressure ventilation (NPPV) is widely used, including in intensive care and home care settings. The success or failure of NPPV depends on how securely the mask fits. Similarly, in continuous positive airway pressure (CPAP) used for sleep apnea syndrome (SAS), adequate mask fitting and patient education are important to maintain effective treatment and good adherence.^1^ For both NPPV and CPAP cases, safe and firm mask fitting is not always easy, even for experienced medical professionals. Without supervision, especially in home care settings, these are the most frequent issues. In general, self‐adjustment is fundamental in home care settings; however, wearing the mask itself can be even more challenging in patients with significant upper limb dysfunction. It becomes even more demanding for patients to adjust masks when they want to, especially in the absence of caregivers. This could result in the patient subsequently giving up NPPV, rather than adopting independent mask fitting strategies and overcoming upper limb dysfunction.

We encountered three cases of the necessity to wear NPPV/CPAP masks and upper limb dysfunction. To the best of our knowledge, no similar studies have been reported previously.

## CASE SERIES

Cases descriptions: (Video 1).

**VIDEO 1:** Actual mask wearing. This video demonstrates the actual procedure of wearing a mask. Case 1, who had a left forearm amputation, prepared a mask configuration that was easy to wear initially. Then, she swung the mask up above her head. Finally, she tightened the cheek belt. In case 2, whose upper limbs were both short and had a limited range of motion in shoulder joints, light blue straps were inserted between head and cheek belts on both sides. She swung up the mask with both hands, adjusted the position, and then attached the magnetic belt. In case 3, the patient did not agree to be videotaped, although she agreed to the written presentation. Therefore, under chronic respiratory care certified nurse instruction, a medical staff member demonstrated wearing the mask using the right hand only as a mock patient with left hemiplegia. The mask was placed over the head with a wrist snap while looking in the mirror.

### Case 1. Left proximal forearm amputation

**FIGURE 1:**
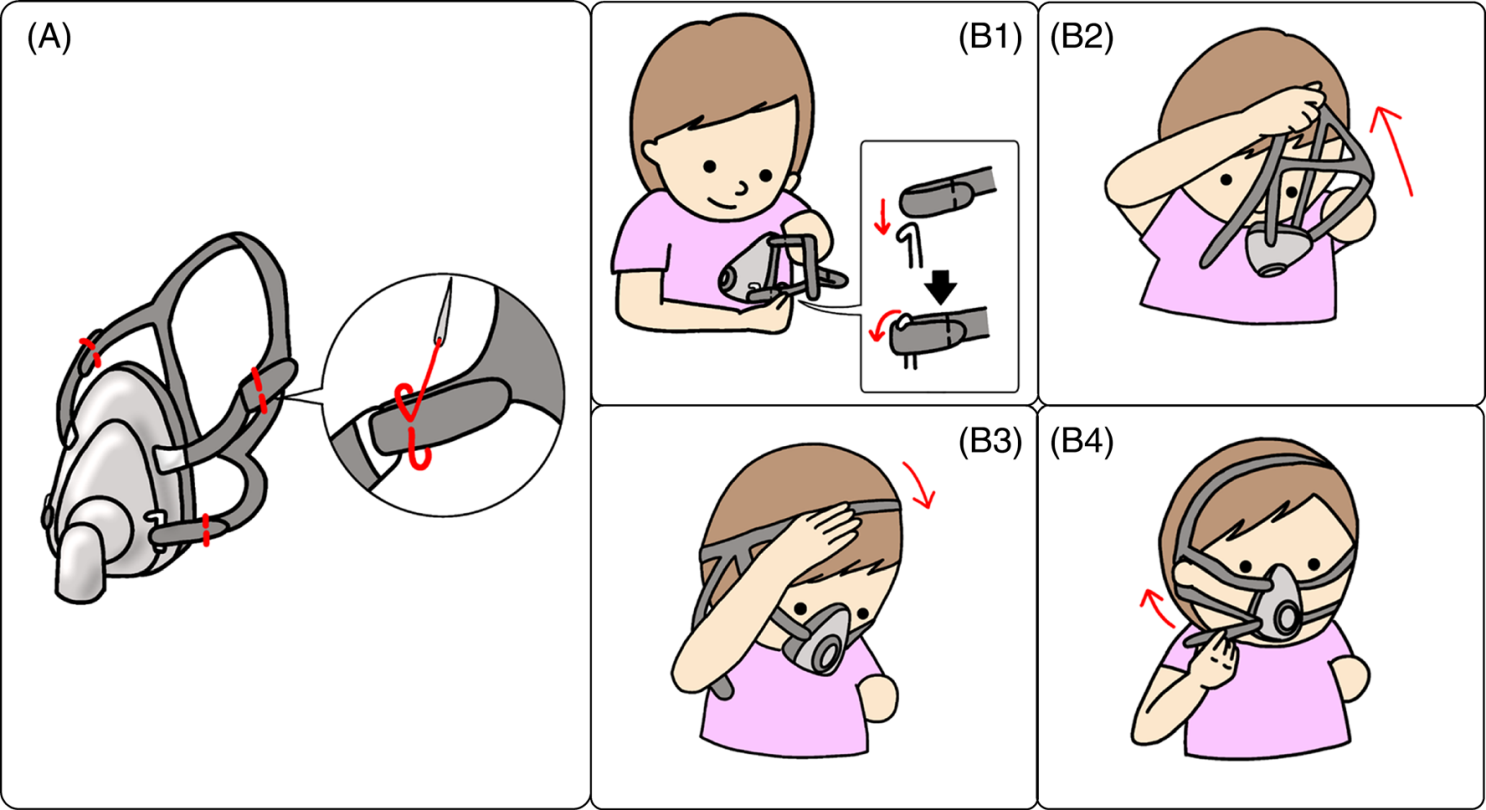
Case 1: Left proximal forearm amputation. (A) Modifications made to the headband. The left forehead and cheek belts and the right forehead belt are sewn and fixed. The sequence in the balloon indicates the procedure for modifying the headband by sewing (the red line is the thread). By hooking the left cheek belt on the mask, a rough overall shape is made. (B) Actual procedure of mask fitting. (B‐1) First, the left cheek belt is hooked onto the mask. The rectangular balloon indicates details. (B‐2) The patient then grasps the headband with the healthy upper limb and swings it up vigorously to put it on. (B‐3) Thereafter, the headband is positioned correctly. (B‐4) Finally, the patient fastens the cheek belt.

A woman in her 50s with a left forearm amputation due to an accident had a medical history of asthma, hypertension, and chronic heart failure. She visited the emergency room almost monthly due to anxiety. She was admitted to our ward due to severe hypercapnic respiratory failure. Following NPPV‐supported improvement, she was diagnosed with obstructive sleep apnea (OSAS) with obesity. She had a high Epworth sleepiness score (ESS) of 16 points, an apnea‐hypopnea index (AHI) of 24.3 points, and the lowest oxygen saturation of 80% during night sleep. We recommended CPAP; however, she lived alone and had no caregiver. Owing to her advanced age and financial situation, she could not be admitted to a facility. Therefore, we formed a multidisciplinary team and trained her to wear a CPAP mask by herself. Thereafter, a chronic respiratory nursing certified nurse (CRCN) taught her how to wear a headband, and stitched the headband for ease of handling (Figure 1) as follows: the headband on the left forehead and cheek, a disabled side, and the headband on the right forehead were sewn and fixed (Mask: ResMed Air Fit F10; ResMed, Inc., San Diego, CA), and the headband on the right cheek, a healthy side, was adjustable. Daily practice was conducted by the ward nurses and occupational therapist (OT), and information was shared among these professionals. Although it took 43 days of hospital stay and 35 days of practice, there were no unscheduled visits after discharge, and the hypercapnia improved significantly.

### Case 2. Short upper limbs

A woman in her 50s with a history of congenital spondyloepiphyseal dysplasia tarda bound to a wheelchair due to short limbs had restrictive ventilatory failure due to barrel chest deformity and was already on home oxygen therapy. Due to daytime somnolence and elevated end‐expiratory carbon dioxide levels as high as 65–70 mmHg, she was admitted to our hospital and NPPV was initiated. Owing to her short upper limbs and restricted range of motion of both shoulder joints, she could not raise her hands above cheek level, making it difficult for her to wear the mask over her head.

**FIGURE 2:**
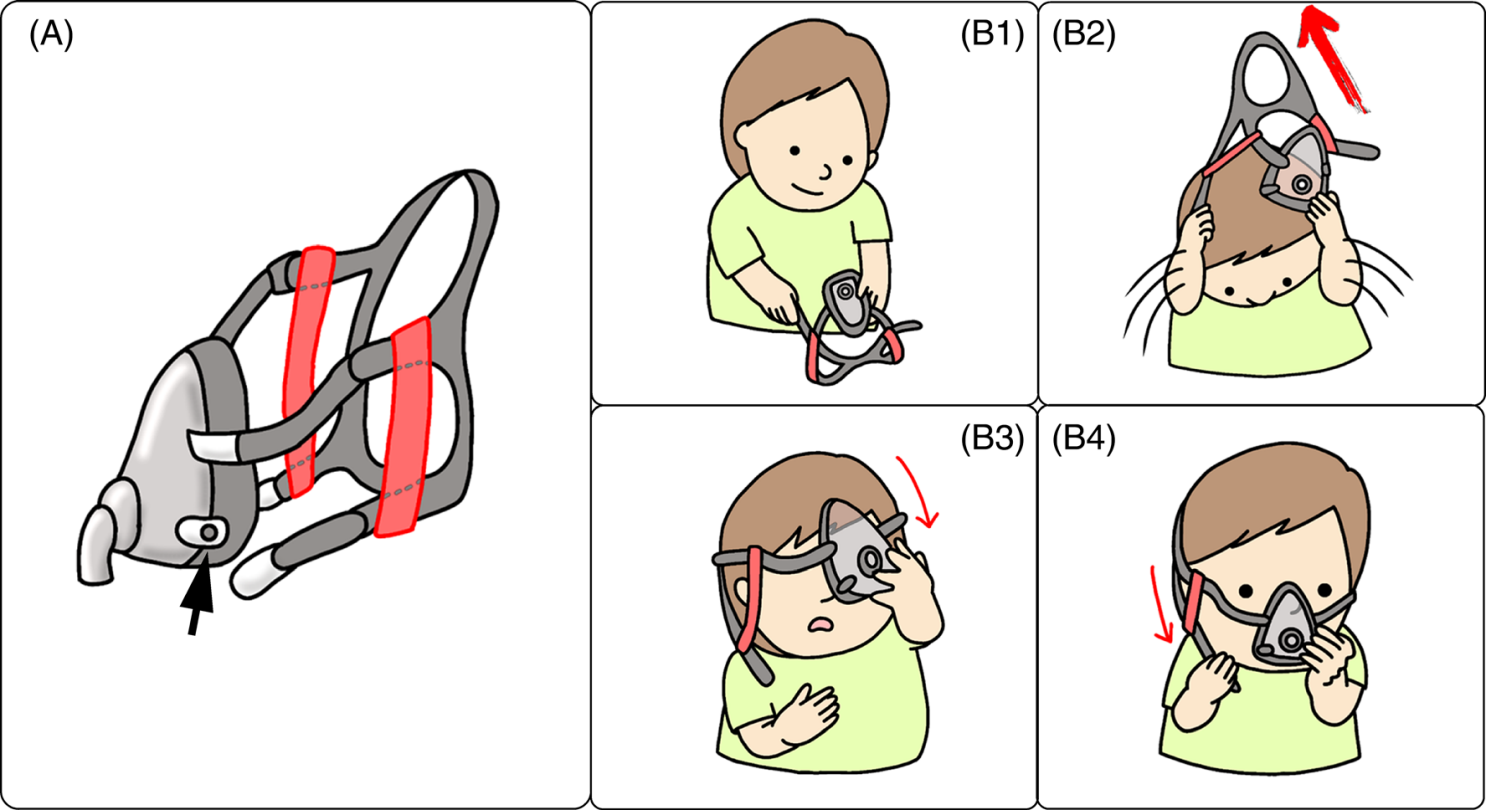
Case 2: Short upper limb. (A) Modifications made to the headband. The soft straps between the forehead belt and cheek belt are added to both sides to maintain the configuration of the headband (red bands). A headband with a magnetic attachment (arrowhead). (B) Actual procedure of mask fitting. (B‐1) First, the patient prepares to throw the mask up by grasping the mask on one side of the cheek belt. (B‐2) Then the patient tilts the head forward and throws the headband with mask vigorously over the head. (B‐3) Thereafter, the patient pulls the mask down and positions it. (B‐4) Finally, the magnets at the end of the cheek belts on both sides are attached to the mask.

Her main caregiver was her elderly mother. However, she wished to live independently at home and we began training her on wearing an NPPV mask on her own. The CRCN adopted a headband with magnetic attachment (ResMed Air Fit F20; ResMed, Inc., San Diego, CA) and sewed the Velcro portion of the size adjuster to allow the mask size to be fixed. Furthermore, the CRCN added straps between the forehead belt and cheek belt on both sides to maintain headband configuration. These additional straps allowed the mask to be placed over the head by swinging up both cheek belts (Figure 2). The ward nurses shared the responsibility of assisting the patient and confirming daily fitting, while OT facilitated adjustment training practice. The training was conducted over 14 days. After discharge, her condition was stable and end‐expiratory carbon dioxide level was between 45 and 55 mmHg.

### Case 3. Left hemiplegia due to cardiogenic cerebral embolism

A woman in her 60s with cardiogenic cerebral infarction was diagnosed with OSAS after a right frontal lobe stroke. This resulted in hemiplegia; however, her condition stabilized. The AHI was 66.6 episodes per hour, and the maximum oxygen desaturation level was 46% during the night. She was transferred to the rehabilitation ward and put on CPAP therapy. Initially, she wore the CPAP mask with the assistance of ward nurses. Thereafter, she reported effectiveness of CPAP therapy as her daytime alertness improved, and she learned to wear the mask by herself.

A CRCN and OT instructed her on fitting the mask as follows: Considering the left hemiplegia, a headband with magnetic attachment (ResMed Air Fit F20; ResMed, Inc., San Diego, CA) was used, and as the left cheek belt remained attached, the right cheek belt was held and thrown over the head with a snap of the wrist while looking in the mirror. Thereafter, after checking the mask position in the mirror again, the magnet on the right cheek belt was attached and the position was adjusted accordingly. The patient received 6 days of training, was discharged 5 weeks after completing her training, and has been effectively using CPAP daily thereafter.

## DISCUSSION

In Japan, the prevalence rate of SAS can be estimated between 17 and 23%, based on the various survey method.^2^, ^3^ SAS is known to cause not only daytime sleepiness or poor concentration, but also complications such as hypertension, atrial fibrillation, and glucose intolerance.^4^, ^5^, ^6^ Among the treatment options, CPAP is the most common and reliable. However, its success depends on accurate evaluation by polysomnography and adequate patient education. After the implementation of CPAP therapy, adherence to the treatment should be reassessed. In case of non‐compliance, appropriate response is required.^7^

Providing instructions on mask fitting is paramount at the beginning of the treatment. In particular, facial skin problems due to mask pressure should be promptly dealt with as they affect the continuation of treatment.^8^, ^9^ However, to the best of our knowledge, there are no studies that focus on upper limb dysfunction and provide detailed instructions in the same case.

Additionally, SAS may be complicated in patients with cerebrovascular disorders, and several studies have been conducted on the effectiveness and adherence to CPAP therapy.^10^, ^11^ However, these reports do not provide clear guidelines about effective mask fitting, especially for patients with hemiplegia who cannot use their upper limbs, as in our case 3. Furthermore, differences in adherence based on the degree of paralysis and site of brain damage have not been investigated. Hsu et al. analysed the relationship between SAS and stroke; however, they excluded those who could not wear the mask on their own or did not have a nighttime caregiver.^11^

When the patient can effectively use both upper limbs, the procedure for fitting the mask entails placing a roughly shaped headband over the head and then adjusting its position and tightness to prevent leakage. However, patients with upper limb dysfunction have difficulty completing these steps. In addition to providing sufficient instruction time, our new approach involved simplifying the wearing procedure and making modifications to the headband. While these additions made fine adjustment difficult, they allowed patients to wear the mask independently even if they were unable to raise both upper limbs above the head.

From an economic point of view, time‐consuming instructions are not well‐received by patients and medical staffs. Circumstances where time‐consuming mask‐fitting instructions are to be provided to patients with upper limb dysfunction have not been discussed in the literature. While some patients can be expected to abandon CPAP/NPPV therapy, those who do not can be expected to benefit from such efforts. Thus, there is a need to map out individualized protocols for different degrees of disability. In this study, we showed examples of how to teach mask‐wearing in three types of functional impairments: proximal forearm amputation, short limb with limited range of motion, and hemiplegia.

A limitation of this study is that while there are multiple causes of upper limb dysfunction, our case series assessed only three cases. Further, these cases were distinct in that the patients had no cognitive dysfunction and had social backgrounds that allowed them to realize their desire for independence. Establishing individualized teaching methods according to the site and degree of impairment was considered a future challenge. Furthermore, it must be clarified which staffing is necessary to complete the instruction in a short time. If a patient's condition worsens and he/she cannot wear a mask by himself/herself, this can lead to a serious problem. Therefore, advance care planning in the worst‐case scenario needs to be considered by the multidisciplinary team as they begin teaching mask‐wearing.

## AUTHOR CONTRIBUTIONS

Tomoko Kuroda, a CRCN, was deeply involved in the instruction of the three patients and implemented mask innovations. Kazushige Ichikawa, an OT, also involved in the instruction of the three patients. Satoshi Hinata, Kaoru Chiba, Ken Okabayashi, and Yuko Asato involved in treatment of these patients in the respiratory ward and outpatient ward. Hiromi Aono and Kazuyoshi Marumo involved in the preparation and supervision of this manuscript.

## CONFLICT OF INTEREST STATEMENT

None declared.

## ETHICS STATEMENT

The authors declare that appropriate written informed consent was obtained for the publication of this manuscript and accompanying images. This study was approved by the Ethics Committee of Tokyo Metropolitan Police Hospital (21‐a09).

## Data Availability

The data that support the findings of this study are available from the corresponding author upon reasonable request.

## References

[1] Mehrtash M , Bakker JP , Ayas N . Predictors of continuous positive airway pressure adherence in patients with obstructive sleep apnea. Lung. 2019;197:115–121. 30617618 10.1007/s00408-018-00193-1

[2] Nakayama‐Ashida Y , Takegami M , Chin K , Sumi K , Nakamura T , Takahashi K , et al. Sleep‐disordered breathing in the usual lifestyle setting as detected with home monitoring in a population of working men in Japan. Sleep. 2008;31:419–425. 18363319 10.1093/sleep/31.3.419 PMC2276741

[3] Suzuki Y , Ikeda A , Wada H , Maruyama K , Miyachi N , Filomeno R , et al. Prevalence of sleep‐disordered breathing among women working in the aged care services in Japan. Int Arch Occup Environ Health. 2019;92:309–316. 30483875 10.1007/s00420-018-1381-9

[4] Salman LA , Shulman R , Cohen JB . Obstructive sleep apnea, hypertension, and cardiovascular risk: epidemiology, pathophysiology, and management. Curr Cardiol Rep. 2020;22:6. 31955254 10.1007/s11886-020-1257-y

[5] Linz D , McEvoy RD , Cowie MR , Somers V , Nattel S , Levy P , et al. Associations of obstructive sleep apnea with atrial fibrillation and continuous positive pressure treatment: a review. JAMA Cardiol. 2018;3:532–540. 29541763 10.1001/jamacardio.2018.0095

[6] Schipper SBJ , Van Veen MM , Elders PJM , van Straten A , Van Der Werf YD , Knutson KL , et al. Sleep disorders in people with type 2 diabetes and associated health outcomes: a review of the literature. Diabetologia. 2021;64:2367–2377. 34401953 10.1007/s00125-021-05541-0 PMC8494668

[7] Bakker JP , Weaver TE , Parthasarathy S , Aloia MS , Adherence to CPAP . What should we be aiming for, and how can we get there? Chest. 2019;155:1272–1287. 30684472 10.1016/j.chest.2019.01.012

[8] Arundel L , Irani E , Barkema G . Reducing incidence of medical device‐related pressure injuries from use of CPAP/BiPAP masks. A quality improvement project. J Wound Ostomy Continence Nurs. 2021;48:108–114. 33690244 10.1097/WON.0000000000000742

[9] Otero DP , Domínguez DV , Femández LH , Magariño AS , González VJ , Klepzing JVG , et al. Preventing facial pressure ulcers in patients under non‐invasive mechanical ventilation: a randomized control trial. J Wound Care. 2017;26:128–136. 28277990 10.12968/jowc.2017.26.3.128

[10] Khot S , Barnett H , Davis A , Siv J , Crane D , Kunze A , et al. Intensive continuous positive airway pressure adherence program during stroke rehabilitation. Stroke. 2019;50:1895–1897. 31104619 10.1161/STROKEAHA.119.024795 PMC9367631

[11] Hsu CY , Vennelle M , Li HY , Engleman HM , Dennis MS , Douglas NJ . Sleep‐disordered breathing after stroke: a randomised controlled trial of continuous positive airway pressure. J Neurol Neurosurg Psychiatry. 2006;77:1143–1149. 16772358 10.1136/jnnp.2005.086686 PMC2077531

